# Effect of scattered megavoltage x‐rays on markerless tumor tracking using dual energy kilovoltage imaging

**DOI:** 10.1002/acm2.13993

**Published:** 2023-04-18

**Authors:** Mandeep Kaur, Jason Luce, Mathias Lehmann, Daniel Morf, Liangjia Zhu, Hyejoo Kang, Michal Walczak, Matthew M. Harkenrider, John C. Roeske

**Affiliations:** ^1^ Department of Radiation Oncology, Stritch School of Medicine, Cardinal Bernadin Cancer Center Loyola University Chicago Maywood Illinois USA; ^2^ Department of Radiation Oncology Loyola University Medical Center Maywood Illinois USA; ^3^ Varian Medical Systems Palo Alto California USA

**Keywords:** dual energy imaging, markerless tumor tracking, megavoltage scatter, wavelet‐FFT

## Abstract

**Purpose:**

To determine the effect of megavoltage (MV) scatter on the accuracy of markerless tumor tracking (MTT) for lung tumors using dual energy (DE) imaging and to consider a post‐processing technique to mitigate the effects of MV scatter on DE‐MTT.

**Methods:**

A Varian TrueBeam linac was used to acquire a series of interleaved 60/120 kVp images of a motion phantom with simulated tumors (10 and 15 mm diameter). Two sets of consecutive high/low energy projections were acquired, with and without MV beam delivery. The MV field sizes (FS) ranged from 2 × 2 cm^2^–6 × 6 cm^2^ in steps of 1 × 1 cm^2^. Weighted logarithmic subtraction was performed on sequential images to produce soft‐tissue images for kV only (DE_kV_) and kV with MV beam on (DE_kV+MV_). Wavelet and fast Fourier transformation filtering (wavelet‐FFT) was used to remove stripe noise introduced by MV scatter in the DE images (${\mathrm{DE}}_{\mathrm{kV}+\mathrm{MV}}^{\mathrm{Corr}}$). A template‐based matching algorithm was then used to track the target on DE_kV,_ DE_kV+MV_, and ${\mathrm{DE}}_{\mathrm{kV}+\mathrm{MV}}^{\mathrm{Corr}}$ images. Tracking accuracy was evaluated using the tracking success rate (TSR) and mean absolute error (MAE).

**Results:**

For the 10 and 15 mm targets, the TSR for DE_kV_ images was 98.7% and 100%, and MAE was 0.53 and 0.42 mm, respectively. For the 10 mm target, the TSR, including the effects of MV scatter, ranged from 86.5% (2 × 2 cm^2^) to 69.4% (6 × 6 cm^2^), while the MAE ranged from 2.05 mm to 4.04 mm. The application of wavelet‐FFT algorithm to remove stripe noise (${\mathrm{DE}}_{\mathrm{kV}+\mathrm{MV}}^{\mathrm{Corr}}$) resulted in TSR values of 96.9% (2 × 2 cm^2^) to 93.4% (6 × 6 cm^2^) and subsequent MAE values were 0.89 mm to 1.37 mm. Similar trends were observed for the 15 mm target.

**Conclusion:**

MV scatter significantly impacts the tracking accuracy of lung tumors using DE images. Wavelet‐FFT filtering can improve the accuracy of DE‐MTT during treatment.

## 1 INTRODUCTION

In radiation therapy of lung cancer, precise localization of the tumor in the presence of breathing motion is vital for treatment accuracy.^1^ Markerless lung tumor tracking using kilovoltage (kV) images has been previously reported, negating the potential complications and cost associated with implanting markers.^2^, ^3^, ^4^, ^5^, ^6^ One of the barriers to markerless tumor tracking (MTT) is the inferior tumor visibility on single energy (SE) kV images in cases where the tumor is obstructed by bony structures.^6^ Our group and others have explored dual energy (DE) kV imaging to address the limitations of MTT using SE images.^7^, ^8^, ^9^, ^10^, ^11^, ^12^ DE imaging involves obtaining kV images at high (i.e., 120 kVp) and low (i.e., 60 kVp) energies followed by weighted logarithmic subtraction to suppress bone. These prior studies indicate the potential of DE imaging to enhance the visualization of lung tumors and provide accurate intra‐fractional imaging for MTT.^8^, ^9^, ^10^

In a clinical scenario, the MV (therapeutic) beam will be delivered while kV images are acquired for MTT. The conventional linear accelerator design has an on‐board imager (OBI) that is located at right angles with respect to the MV beam. During the treatment, scattered x‐rays from the MV beam are incident on the kV detector. This process introduces “horizontal stripe noise” on kV images caused by the accumulation of scatter signal of the MV treatment beam pulses which are delivered asynchronously to the read out sequence of the kV imager rows. This additional scatter signal not only adds beam noise but also introduces horizontal structures in the kV images when the MV beam is on.^13^ Luo et al. demonstrated that MV scatter reduces the contrast‐to‐noise ratio (CNR) of kV images up to 30%.^13^ This contrast degradation, as well as the horizontal line structures introduced by the MV pulses, can cause detection errors or lack of detection of the tumor, making MTT challenging. Hence, there is a clinical need to mitigate the effect of MV stripe noise on kV images to allow for real‐time tumor tracking.

Several solutions have been proposed to improve kV image quality during the MV beam treatment delivery. Van Herk et al. proposed an alternating sequence between the kV pulse and MV pulse such that the kV source was enabled during every other frame. This approach was demonstrated for a volumetric modulated radiotherapy (VMAT) delivery and showed that cone beam computed tomography (CBCT) reconstruction was nearly identical to that obtained without the MV beam, with the exception of some unavoidable noise from MV scatter.^14^ Ling et al. also examined the effect of VMAT delivery on CBCT image quality. They divided each treatment arc control point into two, the first for the MV radiation delivery and the second for acquiring kV projections. This technique repeatedly pauses the MV beam delivery to acquire scatter‐free kV projections. Using this approach, they were able to produce nearly scatter‐free CBCT images, however, clinical implementation would require synchronization of kV and MV beams.^15^ Another approach places emphasis on calculating the MV scatter map.^16^, ^17^ For these studies, three images were typically obtained: (1) kV only images (without MV beam irradiation), (2) concurrent kV images during MV beam irradiation (MV+kV images), and (3) images containing MV‐scatter only (MV‐scatter map). Each MV‐scatter map was separately subtracted from the MV+kV images to obtain MV scatter corrected images.^17^ The image contrast was improved by this scatter measurement‐based correction method. However, this method was not able to eliminate stripe noise from kV images acquired during MV beam irradiation.^17^ An alternative approach using post‐processing techniques may be useful in removing stripes. Several techniques have been proposed in other disciplines including moving average filtering,^18^ interpolation approaches,^19^ frequency filtering with fast Fourier transform (FFT),^20^ as well as approaches using wavelets.^21^, ^22^

The purpose of this study is twofold. First, we experimentally evaluate the impact of MV scatter and subsequent stripe noise on the accuracy of MTT with DE imaging. Second, we investigate a post‐processing technique to remove stripe noise on DE images and determine if this technique is sufficient to reduce the image degradation due to MV scatter. To our knowledge, this is the first study to investigate both concepts related to MTT using DE imaging.

## 2 MATERIALS AND METHODS

### 2.1 Phantom

The CIRS dynamic thorax motion phantom (CIRS Inc. Norfolk, VA) was used in this study. The phantom approximates an average human thorax in size and with the embedded structures (lung cavities, ribs, and spine). Two different spherical target inserts with diameters of 10 and 15 mm were used in this study. A lung equivalent cylinder containing the spherical target was inserted into the lung equivalent lobe of the phantom. Subsequently, the cos^4^ waveform^23^ with amplitude = 30 mm and period = 5 s was applied to produce target motion in the superior‐inferior direction.

### 2.2 Data acquisition

The on‐board imager (OBI) of a TrueBeam linear accelerator (Varian Medical Systems, Palo Alto, CA) was used in Developer mode to acquire fast‐kV switching images for DE imaging. The OBI system consists of a kV x‐ray tube (GS 1542, Varex Imaging, Salt Lake City, UT) with an amorphous‐silicon (*a*Si) flat panel imager (PaxScan 4030CB, Varex Imaging, Salt Lake City, UT) accompanied by an x‐ray generator (EPS 45−80, EMD Technologies, Saint‐Eustache, Quebec). Fast‐kV switching changes voltages between each projection so that the odd (or even) projections correspond to the low (or high) tube voltages at a frame rate of 15 frames per second (fps). A third image (DE) is then produced offline from consecutive high/low image pairs as described in the next section.

For these studies, the phantom was placed on the treatment table with the tumor in its static position located at the isocenter. Two types of images were obtained: (1) kV images without MV beam irradiation for reference (kV only images) and (2) kV images when the MV beam is on (kV+MV images). Both sets of images were acquired over a 180° arc (gantry angles: 360−180 degrees; imaging angles: 90−270 degrees) using fast kV‐switching as discussed above. Note that the difference in the gantry/imaging angles is due to the 90‐degree offset of the OBI with the gantry. The mA setting for each pulse was adjusted to minimize the difference in air exposure between the 60 kVp (60 mA, 20 ms) and 120 kVp (15 mA, 20 ms) acquisitions.^8^, ^9^, ^10^ For the MV irradiations, a 6X‐FFF beam was delivered over the same gantry angles used previously with a dose rate of 1400 MU/min, which is the highest possible dose rate for this energy on TrueBeam, representing the worst case scenario. At this particular dose rate, the MV pulses are delivered with 360 pulses per second, meaning that during the 16 ms required to read out the two halves of the kV imager area, 5−6 MV pulses are delivered. The field size was varied from 2 × 2 cm^2^ to 6 × 6 cm^2^ in steps of 1 × 1 cm^2^ to cover typical field sizes used for lung stereotactic body radiotherapy (SBRT). A summary of the experimental parameters is shown in Table 1.

**TABLE 1:** Summary of experimental parameters

MV energy	6X‐FFF
Field size	2 × 2 to 6 × 6 cm^2^(step sizes of 1 × 1 cm^2^)
SAD	100 cm
SID	150 cm
Dose rate	1400 MU/min
Imaging angles	90−270 degrees
Gantry angles	360−180 degrees
Collimator angle	0 degrees

Abbreviations: SAD, source‐axial distance; SID, source‐to‐imager distance.

### 2.3 Dual energy weighted logarithmic subtraction

To create the DE images, weighted logarithmic subtraction (WLS) was performed offline on paired 60/120 kVp images to reduce bone in the resultant soft‐tissue images.^8^, ^9^, ^10^ WLS was performed to generate DE_kV_ and DE_kV+MV_ as follows^24^:

(1) $$\;DE_{kV\;}\;\;=lnI_{kV}^{H}-\;w_{1\;}lnI_{kV}^{L}$$

(2) $$\;DE_{kV+MV\;}\;=lnI_{kV+MV}^{H}-\;w_{2\;}lnI_{kV+MV}^{L}$$

where $I_{kV}^{H},$ $I_{kV}^{L}$ are the intensities of individual pixels on the high and low energy projections, respectively without MV beam irradiation, while $I_{kV+MV}^{H},$ $I_{kV+MV}^{L}$ are the high and low energy projections, respectively, when the MV beam is on. The weighting factors, *w_1_* and *w_2_*, were used to produce $DE_{kV\;}$ and $DE_{kV+MV\;}$ images. Weighting factor optimization was performed using a custom graphic user interface (GUI) in MATLAB R2022a (MathWorks, Natick, MA, USA) to show the resulting DE images interactively as the weighting factor is manually adjusted in steps of 0.01 until the bone was visually removed and the soft tissue image remained. Once the best weighting factor was set for one DE image, it was applied to the next 20 projections, which corresponded to approximately 13 degrees of imaging. A previous study demonstrated that the optimal weighting factor does not change significantly over this range.^9^ This process was applied to all images acquired over the 180° arc to separately create $DE_{kV\;}$ and $DE_{kV+MV\;}$images.

### 2.4 MV stripe noise removal

**FIGURE 1:**
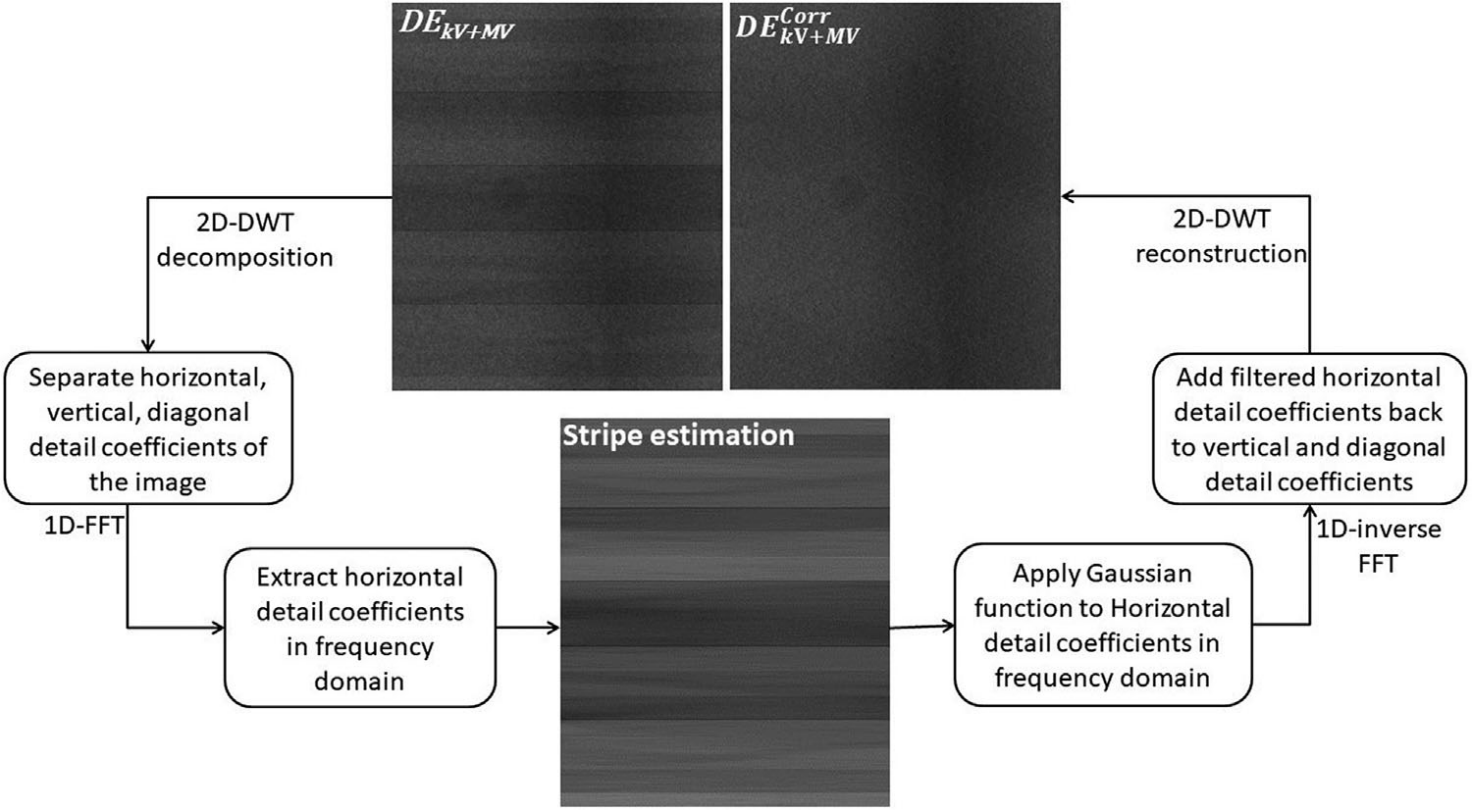
Flowchart of the wavelet‐FFT algorithm for stripe removal.

The core algorithm of combined wavelet and FFT filtering used in the present study was proposed by Münch et al.,^22^ and termed wavelet‐FFT filtering. The algorithm for stripe removal is shown in Figure 1. First, a two‐dimensional discrete wavelet decomposition (2D‐DWT) breaks down the image into three components: diagonal, vertical, and horizontal direction data. The FFT is then used to separate out the horizontal stripes, which are identified by their linear structure in the wavelet. Subsequently, the horizontal coefficients are multiplied by a Gaussian function to eliminate the stripe noise in the frequency domain. The restored image is then generated by applying the 2D‐DWT reconstruction. This multi‐step process eliminates the unwanted stripes from the image while preserving the other image details.

This algorithm was implemented for stripe noise removal using in‐house software in MATLAB R2022a (MathWorks, Natick, MA, USA). The wavelet‐FFT algorithm has several parameters that require optimization, the first of which is the decomposition level (ℓ) to select the frequency band where the filter is applied. We investigated various parameters to find the best combination that produces a visually satisfying result with a minimal amount of stripe artifacts while still preserving the overall image quality. After assessing various parameters, it was determined that a decomposition value of ℓ = 7 and a db43 wavelet offered the most satisfactory outcome for all DE images. Sigma (σ) defines the width of FFT coefficients which are damped within each horizontal detail band. A larger value of σ will filter more stripes from the image but may also remove more image information. To maintain a balance between stripe removal and the desired image structure preservation, σ = 7 (for 2 × 2 cm^2^ and 3 × 3 cm^2^) and σ = 11 (4 × 4 cm^2^–6 × 6 cm^2^) pixels was used for this study. Qualitatively, the selected parameters successfully removed horizontal stripes and improved the overall image quality, making it more suitable for tracking across all MV field sizes. It is important to note that this MV stripe removal method was applied to the entire image.

A serendipitous finding of this study demonstrated that vertical stripe removal also improved the quality of DE images (both with and without stripes). The reason is that the individual high/low energy images constituting the DE image are acquired approximately 67 ms apart during which time the gantry moves ∼0.4°.^9^ A previous study has shown that rigid registration between these images can be used, however there are still some subtle misregistration artifacts that can negatively affect tracking.^9^ We observed that the vertical stripe removal method by Münch et al.^22^ is helpful in removing these artifacts and improves image quality. Therefore, we applied the vertical stripe removal method to both $DE_{kV\;}$ and $DE_{kV+MV\;}$ images. The optimized parameters to correct the misregistration artifacts (ℓ = 10, σ = 10, and a db43 wavelet) were subsequently used.

### 2.5 Markerless tumor tracking

A non‐commercial off‐line research software (RapidTrack v3.0, Varian Medical Systems, Palo Alto, CA) was used for template tracking. This software is based on the work by Mostafavi et al. which tracks the position of the target in kV projections.^25^ In the first step, the RapidTrack‐Planning (RTP) software was used to generate templates from a CT scan. Our departmental CT simulator (Somatom Open AS, Siemens Healthineers, Germany) was used to obtain a CT scan of the phantom reconstructed with a 0.6 mm slice thickness. Next, the Eclipse software (version 15.5, Varian) was used to contour the targets and place the treatment isocenter at the center of the static target. CT images and contours were then imported into RapidTrack Planning v1.12 (Varian) to create the individual templates. Two‐dimensional (2D) reference templates were created for every 1° of gantry rotation. The parameters used to generate these templates were taken from a recent publication.^26^

After creating these templates, for each DE image, the template associated with the imaging angle nearest to the projection was selected. To find the best match between the template and the image, the normalized cross correlation (NCC) of 2D template locations within a specified search window on the image was calculated as a degree of similarity. The NCC was calculated as follows:

(3) $$NCC=\frac{1}{n}\sum_{x,y}\frac{\left(f\left(x,y\right)-\bar{f}\right)\left(t\left(x,y\right)-\bar{t}\right)}{\sigma_{f}\sigma_{t}}$$

where *n* is number of pixels in an image and template, and f(x,y) is the intensity within the search region *f* of the DE image at location xandy. Similarly, t(x,y) is the intensity value for template *t*. The parameters $\bar{f\;}and\;\bar{t}$ denotes the mean value of the DE image and the template, respectively. Last, σ_f_ and σ_t_ are standard deviations over the corresponding regions. The tracked location was determined from the peak value of the NCC match surface.

### 2.6 Tracking metrics

To perform quantitative evaluation of template‐based tumor tracking, two metrics were considered: tracking success rate (TSR) and mean absolute error (MAE) with standard deviations (SDs). The TSR is based on the difference between the actual and tracked locations. A successful tracking event for a particular frame is the difference between the tracked location and ground truth (GT) positions of <2 mm. Absolute error refers to the magnitude of difference between the tracked location and the GT. MAE is calculated as the sum of all these absolute errors, divided by the number of frames as given by:

(4) $$\mathrm{MAE}=\frac{1}{N\;}\sum_{i\;=\;1}^{N}\left|x_{i}-x_{t}\right|$$

where *x_i_* and *x_t_* are actual tracked location and GT, respectively, and *N* is the total number of frames for the particular data set. GT positions were obtained based on the cos^4^ programmed motion function with fixed amplitude (30 mm) and period (5 s).

## 3 RESULTS

**FIGURE 2:**
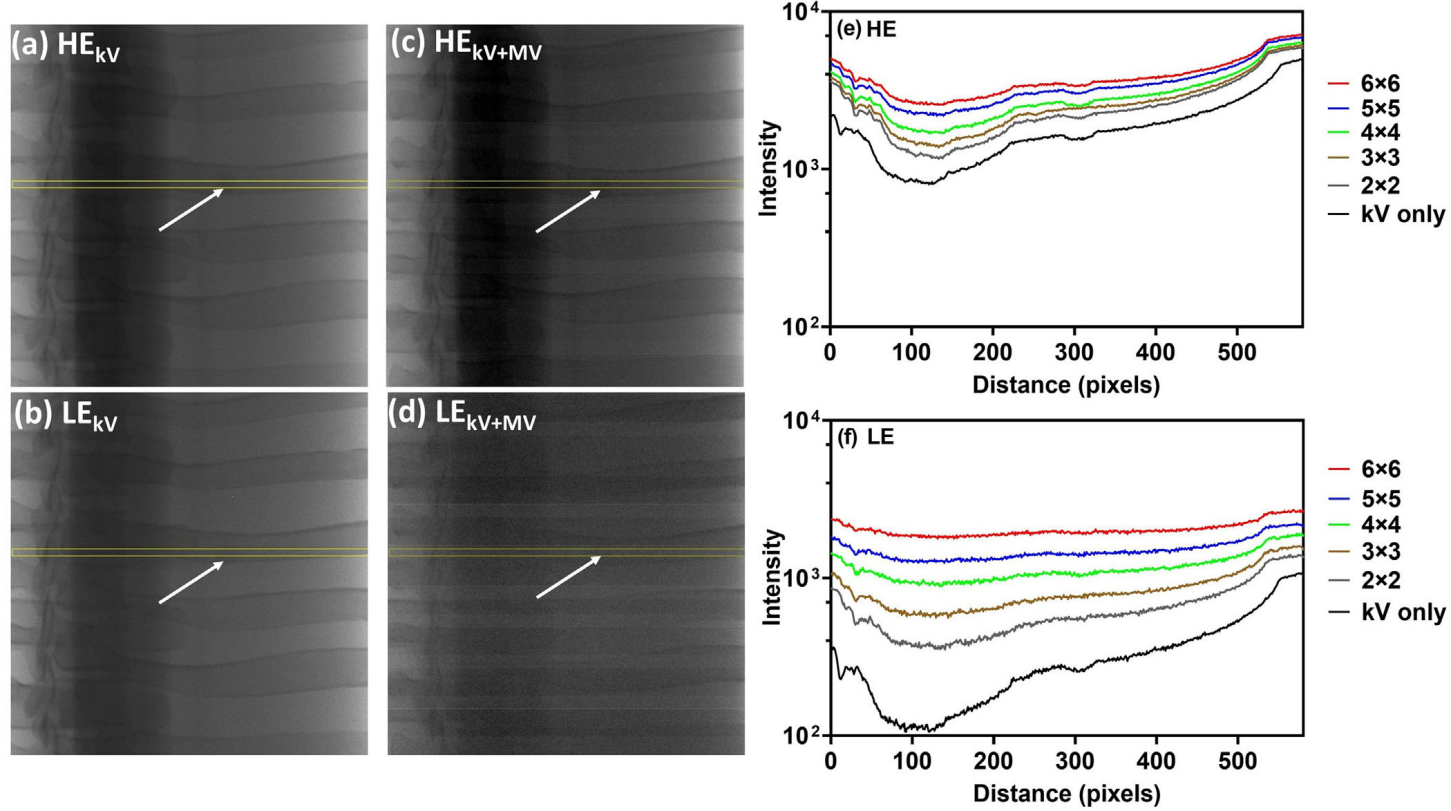
The individual kV images for (a) HE and (b) LE with the MV beam off, (c) HE and (d) LE with MV beam on for 6 × 6 cm^2^ with a 10 mm simulated tumor. Horizontal intensity profiles are shown for HE in (e) and LE in (f). In both cases, a comparison of kV only and kV + MV (FS of 2 × 2 cm^2^–6 × 6 cm^2^) are shown. The yellow rectangle is region of interest (ROI) used to extract corresponding horizontal profile. The white arrow shows the position of the simulated tumor.

The individual images of the high energy (HE) and low energy (LE) kV beams with the MV beam off and on, when a 10 mm target is used, are displayed in Figure 2a–d. By visual inspection, the relative magnitude of the MV stripes is higher in the LE image versus the HE images. Figure 2e,f shows representative intensity profiles through the HE image and the corresponding LE image with (kV+MV) versus without (kV) stripe noise. In both cases, increasing the field size increases the number of counts in the images due to MV scatter accumulation in the row readout of the kV signal. Of note, the effect of MV scatter on the HE images results in a simple vertical shift of the signal. However, on LE images, the MV scatter degrades the image such that much of the signal structure is lost even at field sizes as small as 3 × 3 cm^2^. Although the MV signal is approximately the same in both images, since the kV only signal is significantly lower in the LE image (vs. HE images), the addition of MV scatter overwhelms the kV signal. Therefore, the initial approach to remove stripes on the individual images was not successful for the LE image, since much of the image structure is lost due to MV scatter. As such, we focused on removing stripes on the resultant DE image which provided more consistent results.

**FIGURE 3:**
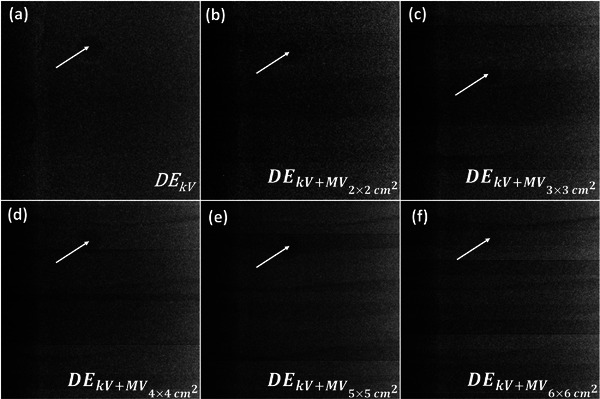
(a) Soft‐tissue DE images generated from paired 60/120 kVp projections when the MV beam is off (DE_kV_) (b)‐(f) Soft‐tissue DE images generated from paired 60/120 kVp projections during MV beam irradiation (DE_kV+MV_) with FS of 2 × 2 cm^2^ to 6 × 6 cm^2^. The white arrow shows the location of the simulated tumor.

Figure 3 shows a region of interest (ROI—8 × 8 cm^2^ around isocenter) of DE_kV_ and DE_kV+MV_ images with a FS of 2 × 2 cm^2^—6 × 6 cm^2^ for a simulated tumor size of 10 mm at a gantry angle of 315.4°. Qualitatively, the stripe noise is intensified with increasing FS, which significantly degrades the quality of DE_kV+MV_ images. The simulated tumor is clearly visible in Figure 3a. However, with increasing MV field size (Figure 3b–f), the target visibility is reduced due to the addition of horizontal stripes, particularly in Figure 3e,f.

**FIGURE 4:**
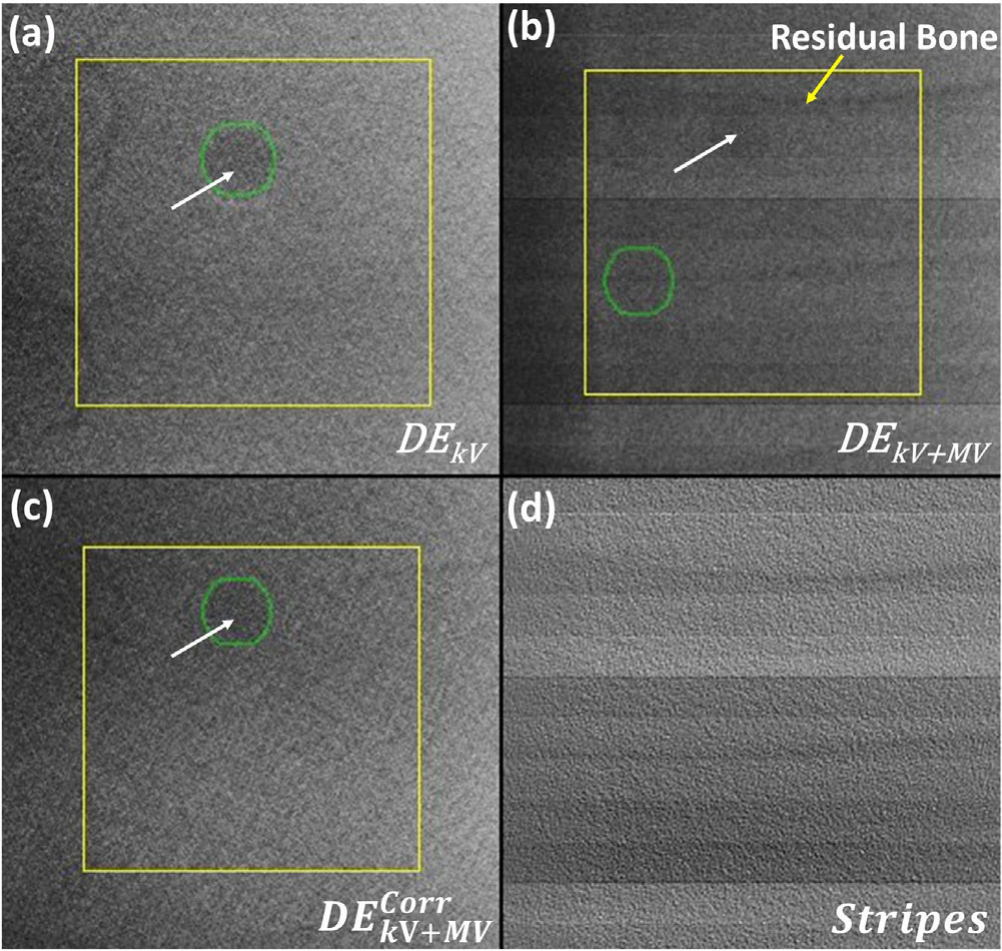
Tracking accuracy comparison of (a) DE images acquired when the MV beam is off (DE_kV_) and (b) DE images acquired during MV beam irradiation (DE_kV+MV_) with a FS of 6 × 6 cm^2^. The corrected DE_kV+MV_ (${\mathrm{DE}}_{\mathrm{kV}+\mathrm{MV}}^{\mathrm{Corr}}$) using the wavelet‐FFT algorithm is shown in (c) while the estimated stripe noise that was removed in shown in (d). The green circle shows the tracked location of the target while the yellow rectangle highlights the search region. The white arrow indicates the location of the simulated tumor. The location of residual bone resulting from slight misregistration of the high and low energy pairs is highlighted in (b) with a yellow arrow.

Figure 4 graphically shows the effect of stripe noise on tracking accuracy. The tracked location (green circle) of the target was correctly overlaid on the DE_kV_ image as shown in Figure 4a_._ The introduction of stripe noise leads the tracking algorithm to misidentify or not be able to locate the target as shown in Figure 4b. Figure 4c shows the restoration of image quality following stripe removal and is similar to that of the reference DE_kV_. Moreover, the simulated tumor was accurately tracked on the ${\mathrm{DE}}_{\mathrm{kV}+\mathrm{MV}}^{\mathrm{corr}}$ image. The additional benefit of this stripe removal algorithm is that it may also further suppress some bone edges (Figure 4b) that were not completely removed by DE processing indicated by the yellow arrow.

**FIGURE 5:**
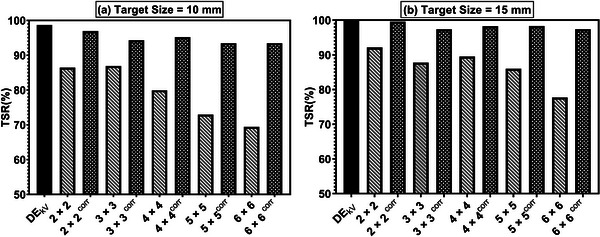
Tracking success rate (TSR) comparison for (a) 10 mm (b) 15 mm for DE_kV_, DE_kV+MV_, and ${\mathrm{DE}}_{\mathrm{kV}+\mathrm{MV}}^{\mathrm{Corr}}$ images using MTT.

Figure 5 shows the TSR for each of the two targets (10 and 15 mm) for DE_kV,_ DE_kV+MV_, and ${\mathrm{DE}}_{\mathrm{kV}+\mathrm{MV}}^{\mathrm{Corr}}$ images. DE_kV_ images have the highest TSR values of 98.7% for the 10 mm target and 100% for the 15 mm target shown by solid black bars. The diagonal pattern bars show the TSR values for the uncorrected images (DE_kV+MV_) as a function of FS. For the 10 mm target, the TSR is reduced to 86% for the 2 × 2 cm^2^ FS. As the FS is increased (i.e., 4 × 4 to 6 × 6 cm^2^), the amount of stripe noise increases (Figure 3) resulting in a more significant reduction in TSR to 69.4%. Similarly for the 15 mm target, DE_kV+MV_ for FS (2 × 2 to 4 × 4 cm^2^) have TSR values of 92.1% to 89.5%, but for the largest FS it decreases to 77.7% as shown in Figure 5b.

The improved tracking accuracy for the corrected images as compared to MV‐scatter images is denoted by the black bars with white dots (Figure 5). Use of the wavelet‐FFT filter on DE_kV+MV_ images was able to increase the TSR to 96.9% for 2 × 2 cm^2^ FS and 94.3% for 3 × 3 cm^2^ FS for the 10 mm simulated tumor. For the larger FS, it was able to accurately track almost 24% more frames as compared to DE_kV+MV_ images (Figure 5a). A similar observation was made for the 15 mm simulated tumor in Figure 5b. For larger FSs, the stripe removal algorithm requires a higher level of wavelet decomposition that not only removes stripes but may also remove some other features in the image. However, greater improvements were observed for larger FSs, that is, the TSR is >93% for 6 × 6 cm^2^ FS for both 10 and 15 mm targets. Of note, in a clinical scenario, 10 mm tumors will typically be treated with FSs between 3 × 3 cm^2^ and 4 × 4 cm^2^, while 15 mm tumors will likely be treated with FSs between 4 × 4 cm^2^ and 5 × 5 cm^2^. For these field sizes, the wavelet‐FFT algorithm worked well to remove the stripes, without significantly impacting tracking accuracy. Subsequently, the TSR for both cases were very close to that of DE_kV_ alone and provided a significant improvement in tracking accuracy as compared to DE_kV+MV_ images.

**FIGURE 6:**
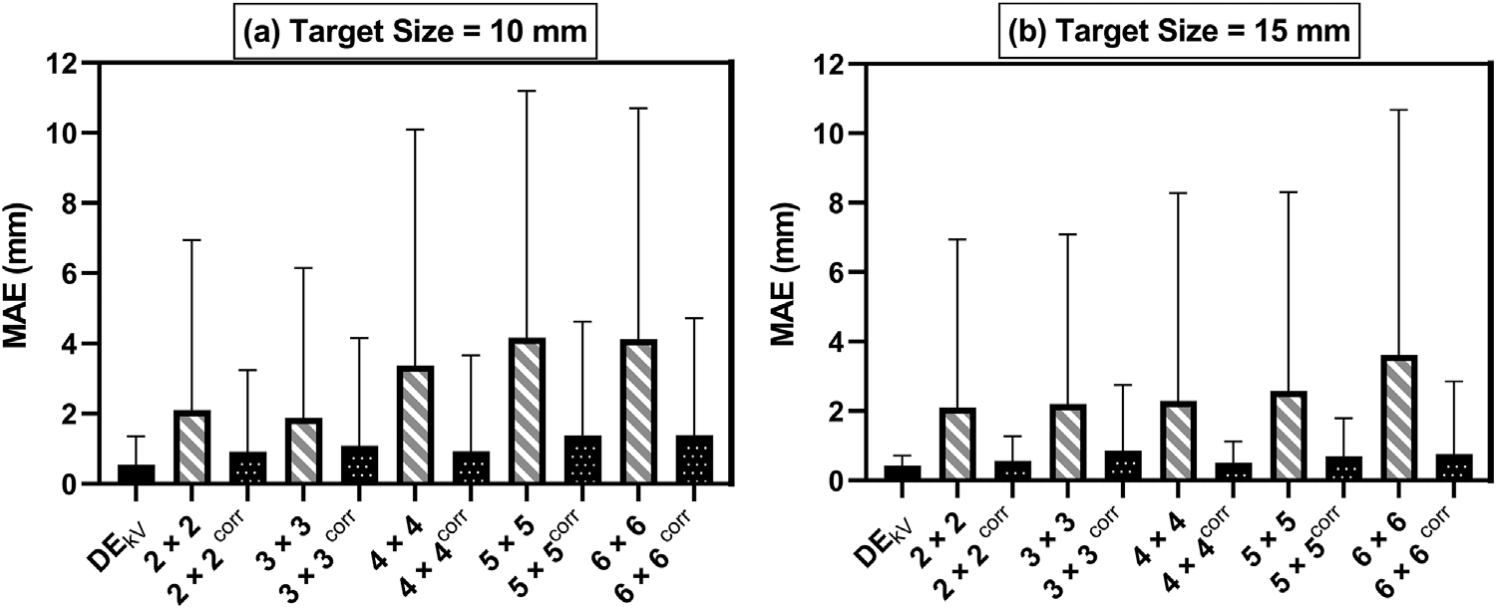
Mean absolute error (MAE) comparison for DE_kV,_ DE_kV+MV_ and $DE_{kV+MV}^{Corr}$ images for 10 and 15 mm targets. The standard deviations of the MAE values for each image type are also shown as bars.

The MAE of the tracked frames with SDs for DE_kV_, DE_kV+MV,_ and ${\mathrm{DE}}_{\mathrm{kV}+\mathrm{MV}\;}^{\mathrm{corr}}$images are presented in Figure 6. The MAEs of the DE_kV+MV_ images were higher than those of the reference image (DE_kV_) for all the FSs. DE_kV+MV_ images with the largest FSs have the largest MAE values, that is, 4.04 mm for 10 mm target and 3.53 mm for 15 mm target, along with the highest SDs of 6.50 mm and 7.06 mm, respectively. ${\mathrm{DE}}_{\mathrm{kV}+\mathrm{MV}}^{\mathrm{corr}}$ images had lower MAE values as compared to DE_kV+MV_ images over all field sizes for both targets. The most significant improvements were observed with the largest field sizes. For example, the MAE for ${\mathrm{DE}}_{\mathrm{kV}+\mathrm{MV}}^{\mathrm{corr}}$ is four times lower than that of DE_MV+kV_ for 15 mm target for the 6 × 6 cm^2^ FS. A Wilcoxon *t*‐test was performed to compare the MAE between the DE_kV+MV_ and ${\mathrm{DE}}_{\mathrm{kV}+\mathrm{MV}}^{\mathrm{Corr}}$ images over all FSs for both targets. The results revealed a statistically significant reduction in MAE after applying the wavelet‐FFT algorithm, with *p* < 0.05 for all FSs.

## 4 DISCUSSION

In the present study, the quantification and correction of stripe noise on DE images due to MV scatter on the kV imager was analyzed. Previous experience with fast‐kV switching acquisitions and reconstruction of DE images have shown improved tumor tracking compared with conventional (SE) imaging.^7^, ^8^, ^9^, ^10^, ^11^, ^12^ However, these studies focused on the DE image quality for tumor tracking without MV beam irradiation. Hence, prior to clinical implementation, we need to quantify the effect of MV scatter/stripe noise on DE tumor tracking. Our results demonstrate that MV stripe noise decreased the TSR and increased the MAE relative to DE images obtained without MV irradiation. The wavelet‐FFT algorithm was successfully able to remove stripe noise with the smallest MV field sizes having TSR and MAE values closest to DE_kV_ images.

Other approaches to address the MV scatter issue have been presented in the literature, such as the work of Ling et al., which proposed dividing gantry control points into two parts: one for MV‐radiation delivery and the second for acquiring kV images.^14^ This method, however, has the limitation of prolonging the treatment delivery time. Van Herk et al. proposed an alternative technique, which used alternating kV imaging and MV treatment beam delivery to avoid the MV scatter; this resulted in a reduction in the overall number of imaging frames.^14^, ^15^ Results similar to ours were obtained, in that both correction methods resulted in the improved contrast‐to‐noise ratio (CNR) relative to kV‐only CBCT images, but the signal was not completely recovered. Additionally, both studies^14^, ^15^ utilized flattened‐filter (FF) MV beams. However, due to the higher dose rate, FFF beams are more suitable for SBRT. Unfortunately, the above‐mentioned techniques are not highly practical for FFF beams due to the high pulse rate. Iramina et al. showed that the MV scatter value factor for FFF beams was larger than that of FF beams.^17^ Their method used scatter maps to decrease the effect of MV scatter, but the stripe noise introduced was not completely eliminated when utilizing FFF beams.^17^

In comparison to these prior studies, the post‐processing algorithm presented in this study was able to remove most stripe noise and resulted in improved tracking accuracy as compared to uncorrected images. The present method improved the tumor tracking accuracy with TSR > 90% for both the targets across all FSs as shown in Figure 5. For smaller field sizes, the TSR was > 95% for both targets, which is comparable to tracking results obtained in a recent study.^27^ An advantage of this technique is that it does not require additional hardware or modifications to the treatment unit. Moreover, this approach was able to remove the stripe noise from the DE images while using FFF beams.

Our eventual goal is to clinically implement MTT for real‐time tracking during MV beam irradiation with minimal image degradation. Further studies and improvements are needed for this technique before it can be implemented clinically. Based on our current and prior results, the tracking accuracy is generally higher for larger versus smaller targets.^9^, ^10^ Therefore, the tracking accuracy of different types of tumors (size, shape, and density) needs to be explored relative to the interplay with the MV beam. As discussed previously, the implemented stripe removal algorithm was able to restore the image quality close to that of the DE_kV_ images for the smallest FS. However, this method may require some refinement to recover the image quality for larger FS to improve tracking accuracy during treatment delivery. Future studies will also consider hardware techniques combined with the present algorithm to mitigate MV scatter on kV images to improve MTT. Lastly, all the images were processed offline. Clinical implementation will require synchronous processing with limited latency. The computational time to implement the stripe removal algorithm in clinical practice plays an important role. A benefit of the wavelet‐FFT algorithm is that it is computationally efficient.^22^ Moreover, this algorithm can be parallelized on GPUs and hence can be performed in near‐real time during treatment delivery.^28^

## 5 CONCLUSION

The present study considered the issue of MV scatter and its effects on the tracking accuracy of simulated lung tumors when using DE imaging. Our results demonstrated that stripe noise, introduced by MV scatter, degrades DE image quality, and reduces target tracking accuracy, relative to images obtained without MV scatter. The addition of a wavelet‐FFT filter, as a post‐processing technique, removes this stripe noise and restores the tracking accuracy to values that are comparable to those obtained without MV scatter.

## AUTHOR CONTRIBUTIONS

All listed authors contributed to the work and to writing the article.

## CONFLICT OF INTEREST STATEMENT

Mathias Lehmann, Daniel Morf, Liangjia Zhu, and Michal Walczak are employed by Varian Medical Systems.

## References

[1] Keall PJ , Mageras GS , Balter JM , et al. The management of respiratory motion in radiation oncology report of AAPM Task Group 76. Med Phys. 2006;33(10):3874‐3900. doi: 10.1118/1.2349696 17089851

[2] Lewis JH , Li R , Watkins WT , et al. Markerless lung tumor tracking and trajectory reconstruction using rotational cone‐beam projections: a feasibility study. Phys Med Biol. 2010;55(9):2505‐2522. doi: 10.1088/0031-9155/55/9/006 20393232

[3] Yang Y , Zhong Z , Guo X , et al. A novel markerless technique to evaluate daily lung tumor motion based on conventional cone‐beam CT projection data. Int J Radiat Oncol Biol Phys. 2012;82(5):e749‐e756. doi: 10.1016/j.ijrobp.2011.11.035 22330989

[4] van Sörnsen de Koste JR , Dahele M , Mostafavi H , et al. Markerless tracking of small lung tumors for stereotactic radiotherapy. Med Phys. 2015;42(4):1640‐1652. doi: 10.1118/1.4914401 25832054

[5] Rozario T , Bereg S , Yan Y , et al. An accurate algorithm to match imperfectly matched images for lung tumor detection without markers. J Appl Clin Med Phys. 2015;16(3):131‐140. doi: 10.1120/jacmp.v16i3.5200 26103480 PMC5690140

[6] Shieh CC , Keall PJ , Kuncic Z , Huang CY , Feain I . Markerless tumor tracking using short kilovoltage imaging arcs for lung image‐guided radiotherapy. Phys Med Biol. 2015;60(24):9437‐9454. doi: 10.1088/0031-9155/60/24/9437 26583772 PMC4833659

[7] Hoggarth MA , Luce J , Syeda F , et al. Dual energy imaging using a clinical on‐board imaging system. Phys Med Biol. 2013;58(12):4331‐4340. doi: 10.1088/0031-9155/58/12/4331 23732651

[8] Patel R , Panfil J , Campana M , et al. Markerless motion tracking of lung tumors using dual‐energy fluoroscopy. Med Phys. 2015;42(1):254‐262. doi: 10.1118/1.4903892 25563265

[9] Haytmyradov M , Mostafavi H , Wang A , et al. Markerless tumor tracking using fast‐kV switching dual‐energy fluoroscopy on a benchtop system. Med Phys. 2019;46(7):3235‐3244. doi: 10.1002/mp.13573 31059124 PMC6625841

[10] Roeske JC , Mostafavi H , Haytmyradov M , et al. Characterization of markerless tumor tracking using the on‐board imager of a commercial linear accelerator equipped with fast‐kV switching dual‐energy imaging. Adv Radiat Oncol. 2020;5:1006‐1013. 33089019 10.1016/j.adro.2020.01.008 PMC7560565

[11] Dhont J , Verellen D , Poels K , et al. Feasibility of markerless tumor tracking by sequential dual‐energy fluoroscopy on a clinical tumor tracking system. Radiother Oncol. 2015;117(3):487‐490. doi: 10.1016/j.radonc.2015.08.021 26344088

[12] Menten MJ , Fast MF , Nill S , Oelfke U . Using dual‐energy x‐ray imaging to enhance automated lung tumor tracking during real‐time adaptive radiotherapy. Med Phys. 2015;42(12):6987‐6998. doi: 10.1118/1.4935431 26632054

[13] Luo W , Yoo S , Wu QJ , Wang Z , Yin FF . Analysis of image quality for real‐time target tracking using simultaneous kV‐MV imaging. Med Phys. 2008;35(12):5501‐5509. doi: 10.1118/1.3002313 19175109

[14] van Herk M , Ploeger L , Sonke JJ . A novel method for megavoltage scatter correction in cone‐beam CT acquired concurrent with rotational irradiation. Radiother Oncol. 2011;100(3):365‐369. doi: 10.1016/j.radonc.2011.08.019 21924785

[15] Ling C , Zhang P , Etmektzoglou T , et al. Acquisition of MV‐scatter‐free kilovoltage CBCT images during RapidArc™ or VMAT. Radiother Oncol. 2011;100(1):145‐149. doi: 10.1016/j.radonc.2011.07.010 21821301

[16] Boylan CJ , Marchant T , Stratford J , et al. A megavoltage scatter correction technique for cone‐beam CT images acquired during VMAT delivery. Phys Med Biol. 2012;57:3727‐3739. 22617805 10.1088/0031-9155/57/12/3727

[17] Iramina H , Nakamura M , Miyabe Y , et al. Quantification and correction of the scattered x‐rays from a megavoltage photon beam to a linac‐mounted kilovoltage imaging subsystem. BJR Open. 2020;2(1):20190048. doi: 10.1259/bjro.20190048 33324865 PMC7731796

[18] Boin M , Haibel A . Compensation of ring artefacts in synchrotron tomographic images. Opt Express. 2006;14(25):12071‐12075. doi: 10.1364/OE.14.012071 19529634

[19] Rakwatin P , Takeuchi W , Yasuoka Y . Stripe noise reduction in MODIS data by combining histogram matching with facet filter. IEEE Trans Geosci Remote Sens. 2007;45(6):1844‐1856. doi: 10.1109/TGRS.2007.895841

[20] Arrell K , Wise S , Wood J , Donoghue DNM . Spectral filtering as a method of visualising and removing striped artefacts in digital elevation data. Earth Surf Process Landf. 2008;33:943‐961.

[21] Tang X , Ning R , Yu R , Conover D . Cone beam volume CT image artifacts caused by defective cells in x‐ray flat panel imagers and the artifact removal using a wavelet‐analysis‐based algorithm. Med Phys. 2001;28(5):812‐825. doi: 10.1118/1.1368878 11393477

[22] Münch B , Trtik P , Marone F , Stampanoni M . Stripe and ring artifact removal with combined wavelet—fourier filtering. Opt Express. 2009;17(10):8567‐8591. doi: 10.1364/OE.17.008567 19434191

[23] Lujan AE , Larsen EW , Balter JM , Ten Haken RK . A method for incorporating organ motion due to breathing into 3D dose calculations. Med Phys. 1999;26(5):715‐720. doi: 10.1118/1.598577 10360531

[24] Brody WR , Butt G , Hall A , Macovski A . A method for selective tissue and bone visualization using dual energy scanned projection radiography. Med Phys. 1981;8(3):353‐357. doi: 10.1118/1.594957 7033756

[25] Mostafavi H , Jeung A , Sloutsky A . WE‐A‐134‐07: rapidTrack: a versatile tool for template‐based target tracking during radiotherapy. Med Phys. 2013;40(6Part28):470.

[26] Kaur M , Wagstaff P , Mostafavi H , et al. Effect of different noise reduction techniques and template matching parameters on markerless tumor tracking using dual‐energy imaging. J Appl Clin Med Phys. 2022;23:13821‐13828. doi: 10.1002/acm2.13821 PMC9797162 36350280

[27] Mueller M , Poulsen P , Hansen R , et al. The markerless lung target tracking AAPM Grand Challenge (MATCH) results. Med Phys. 2022;49(2):1161‐1180. doi: 10.1002/mp.15418 34913495 PMC8828678

[28] Bernabé G . Parallel 3D fast wavelet transform comparison on CPUs and GPUs. Ann Multicore GPU Program. 2010;1(1):1101‐1110. doi: 10.1016/j.procs.2010.04.122

